# Comprehensive Analysis of the Systemic Transcriptomic Alternations and Inflammatory Response during the Occurrence and Progress of COVID-19

**DOI:** 10.1155/2021/9998697

**Published:** 2021-08-26

**Authors:** Shaocong Mo, Leijie Dai, Yulin Wang, Biao Song, Zongcheng Yang, Wenchao Gu

**Affiliations:** ^1^Shanghai Medical College, Fudan University, Shanghai, China; ^2^Peking Union Medical College Hospital, Beijing, China; ^3^Department of Implantology, School and Hospital of Stomatology, Cheeloo College of Medicine, Shandong University & Shandong Key Laboratory of Oral Tissue Regeneration & Shandong Engineering Laboratory for Dental Materials and Oral Tissue Regeneration, Jinan, Shandong, China; ^4^Department of Diagnostic Radiology and Nuclear Medicine, Gunma University Graduate School of Medicine, Maebashi, Japan

## Abstract

The pandemic of the coronavirus disease 2019 (COVID-19) has posed huge threats to healthcare systems and the global economy. However, the host response towards COVID-19 on the molecular and cellular levels still lacks full understanding and effective therapies are in urgent need. Here, we integrate three datasets, GSE152641, GSE161777, and GSE157103. Compared to healthy people, 314 differentially expressed genes were identified, which were mostly involved in neutrophil degranulation and cell division. The protein-protein network was established and two significant subsets were filtered by MCODE: ssGSEA and CIBERSORT, which comprehensively revealed the alternation of immune cell abundance. Weighted gene coexpression network analysis (WGCNA) as well as GO and KEGG analyses unveiled the role of neutrophils and T cells during the progress of the disease. Based on the hospital-free days after 45 days of follow-up and statistical methods such as nonnegative matrix factorization (NMF), submap, and linear correlation analysis, 31 genes were regarded as the signature of the peripheral blood of COVID-19. Various immune cells were identified to be related to the prognosis of the patients. Drugs were predicted for the genes in the signature by DGIdb. Overall, our study comprehensively revealed the relationship between the inflammatory response and the disease course, which provided strategies for the treatment of COVID-19.

## 1. Introduction

The global pandemic of the coronavirus disease 2019 (COVID-19), caused by the severe acute respiratory syndrome coronavirus 2 (SARS-CoV-2), exhibits high levels of mortality and morbidity and has posed huge threats to healthcare systems and the global economy [1, 2]. The current COVID-19 pandemic is unprecedented; globally, there have been over 108 million confirmed cases of COVID-19 that have led to over 2.37 million deaths, released by the World Health Organization (WHO) on February 14, 2021 (https://www.worldometers.info/coronavirus/). It is urgent to understand the molecular mechanisms of COVID-19 and identify the patients' susceptibilities so as to find therapeutic interventions.

SARS-CoV-2 belongs to the family of single-stranded RNA viruses known as coronavirus. Its cellular entry requires angiotensin-converting enzyme 2 (ACE2) and transmembrane protease serine 2 (TMPRSS2) for membrane fusion or through the endosomal pathway to infect the host [3–5]. With an oxidative stress and excessive inflammatory response, COVID-19 is being regarded as a systemic disease. With diverse clinical manifestations, COVID-19 patients may present as asymptomatic, with mild respiratory tract infection, acute respiratory distress syndrome, respiratory failure, or even death [6–8]. The imbalance of host immune response and the activation of inflammatory cytokines are called “cytokine storm,” which is related to the severity of the disease and poor prognosis [9].

So far, several immunological characteristics of COVID-19 patients have been demonstrated. Serum c-reactive protein (CRP) and interleukin-6 (IL-6) will increase, while CD4+ and CD8+ T lymphocytes decrease [10–12]. Elevated levels of other inflammatory cytokines and chemokines such as interleukin-2 (IL-2) and interleukin-8 (IL-8), accompanied by increased neutrophils and eosinophils, may also lead to abnormal immune function in COVID-19 patients, further causing more immune cells to be activated and recruited into the lungs, causing “cytokine release syndrome” (CRS) [13–15]. The ratio of macrophages and CD14+ monocytes in PBMC increased, especially in patients with severe COVID-19 in the disease progression stage [16]. At the same time, the number of B cells in the peripheral blood of patients with severe COVID-19 increased significantly but the number of T cells and DC decreased [17]. With a lower baseline levels and functionally exhausted in CD8+ T cells and NK cells, the imbalance of patients in the intensive care unit (ICU) is more prominent [18]. Inflammation is further aggravated by the activation of humoral immunity and the complement system, and the weakening of some classical immune negative signals exacerbates inflammation [9, 19, 20].

Furthermore, several analyses of the transcriptome with high throughput have been conducted to identify the molecular signature of COVID-19 patients [21–25]. However, different studies may have distinct results due to the cohort size and sample heterogeneity. In our study, we aimed to integrate different high-throughput studies to unveil the transcriptomic alterations and differences of immune cell infiltration in the peripheral blood of COVID-19 patients. We uncovered the differentially expressed genes between the healthy people and patients, as well as the DEGs between non-ICU and ICU patients, which underwent comprehensive functional annotation and PPI network construction. We applied ssGSEA and CIBERSORT to evaluate the immune cell infiltration, and the DGIdb database was utilized to predict the drug-gene interaction. By profiling the characteristics of COVID-19 patients with different courses, we hoped to provide new insights into molecular pathogenesis and potential therapeutic targets of COVID-19.

## 2. Materials and Methods

**Figure 1:**
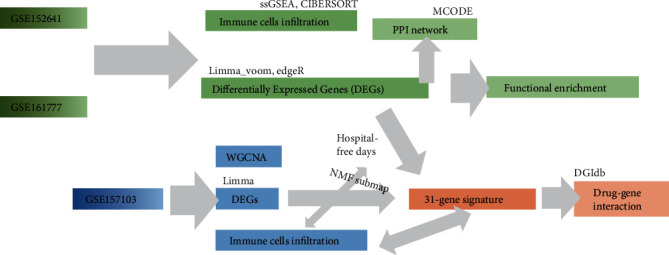
Workflow in the present study.

The workflow of the study was shown in Figure 1.

### 2.1. Data Processing

Two gene expression series, GSE152641 and GSE161777 [26, 27], which contained blood samples from healthy controls and patients, were downloaded from the Gene Expression Omnibus (GEO) (https://www.ncbi.nlm.nih.gov/geo/) database on January 3^rd^ and February 23 publicly. GSE152641 contained RNA sequencing data from 62 COVID-19 patients and 24 healthy controls in the form of count. 27 samples of GSE161777 were selected in the form of count, including 13 patients (the first blood sample collection after diagnosis) and 14 healthy controls. Furthermore, GSE157103 [28], another RNA-seq profile in the form of TPM (trans per million), containing peripheral blood leukocyte samples as well as various clinical information from 50 ICU and 50 non-ICU COVID-19 patients was also downloaded from the GEO database publicly for further exploration on January 26.

### 2.2. Identification of Differentially Expressed Genes (DEGs)

The limma [29], limma_voom [30], and edgeR [31] package of R were employed to perform the identification of DEGs; the first one was for data in the TPM format and the latter two were for data in the count format. We consider genes with ∣log_2_ fold change (FC) | >1 and an adjusted *p* value < 0.05 is differentially expressed between two groups. These genes were counted and included in the Venn diagram by the Venndiagram [32] package of R to distinguish the repeated ones.

### 2.3. Pathway and Functional Enrichment Analyses

The clusterProfiler [33] package of R was applied to perform the pathway and functional enrichment analyses, based on the Gene Ontology (GO) database [34] (http://geneontology.org/) and Kyoto Encyclopedia of Genes and Genomes (KEGG) database [35] (https://www.genome.jp/kegg/). GO is a platform constructed from the cellular component (CC), molecular function (MF), and biological process (BP). KEGG is a database widely used to carry out the biological pathway enrichment. Reactome [36] enrichment and UniProt [37] database annotation are directly available on Search Tool for the Retrieval of Interacting Genes/Proteins (STRING, http://string.embl.de/) online database for further functional and pathway enrichment analyses.

### 2.4. Protein-Protein Interaction (PPI) Network Generation and MCODE Analysis

The Search Tool for the Retrieval of Interacting Genes/Proteins (STRING) was also exploited to generate a protein-protein interaction (PPI) network, for the online biological database is based on known and potential protein-protein interaction [38]. Only those genes with interaction scores higher than 0.7 would be picked up and put into Cytoscape software [39] for further visualization analysis. The plug-in Molecular Complex Detection (MCODE) [40] was designed to seek subnets of PPI networks from the STRING online database, and we set all the parameters to default to identify significant subnets.

### 2.5. Evaluation of Immune Cell Abundance

Single-sample gene set enrichment analysis (ssGSEA) was applied to quantify the abundance of infiltration of different types of immune cells through the GVSA [41] package of R. For every single sample, we conducted standardization in order of the gene expression amount and calculated the enrichment scores (ES) by empirical cumulative distribution function, which can finally be transformed into the abundance of infiltration of 28 types of immune cell, and the immune cells gene sets were obtained from a recent study [42]. CIBERSORT [43] (https://cibersort.stanford.edu/), an analytical tool (R script version was utilized) which can estimate the abundances of certain cell types in a mixed cell population, was employed to reveal the proportion of 22 types of immune cells.

### 2.6. Weighted Gene Coexpression Network Analysis (WGCNA)

Weighted gene coexpression network analysis (WGCNA) was aimed at seeking for coexpressed gene modules and exploring the connection between gene networks and the traits being studied. First, according to the expression of genes in different samples, the correlation between any two genes, calculated by Pearson correlation analysis [44], was collected to form a similarity matrix. At the same time, the topological overlap matrix (TOM) method was employed to take both direct and indirect relationships into account. Then, the hierarchical cluster tree would generate whose division of gene modules was based on the TOM value between genes [45]. The module with the highest correlation with sample characteristics was selected for further analysis.

### 2.7. Clustering and Subclass Mapping

Nonnegative matrix factorization (NMF) clustering was conducted by the NMF package in R [46]. Briefly, the best number of clusters was chosen according to the cophenetic value. Then, NMF was conducted with the best rank and the method set to “brunet.” Submap [47] in the GenePattern online tool (https://cloud.genepattern.org/gp) was applied to evaluate the similarities between the clusters identified by NMF and the clinical traits. Patients were classified into 4 groups (divided by the median and the upper and lower quantiles) according to the hospital-free days, which were named B1, B2, B3, and B4. The *p* value in the result was corrected by the Bonferroni method.

### 2.8. Drug-Gene Interaction Prediction

The open-source database named the Drug Gene Interaction Database (DGIdb, https://dgidb.genome.wustl.edu) [48] was utilized to show the known or potential interaction between drugs and genes by entering a list of genes. DGIdb covers over 100000 drug-gene interactions and 42 potentially druggable gene categories involving more than 40000 kinds of genes and 10000 types of drugs, based on PharmGKB, DrugBank, Chembl, TTD, Drug Target Commons, and others. Here, we only included the drug which had been approved and had a certain interaction (activator or inhibitor) with the gene. Then, the interaction network downloaded was visualized by Cytoscape.

### 2.9. Statistical Analysis

The Wilcoxon test was applied to judge whether a statistically significant difference exists among groups. Pearson correlation analysis was employed to conduct the correlation analysis in WGCNA and Spearman correlation coefficient to evaluate the correlation between genes, immune cells, and hospital-free days. All of these statistical analysis were performed in R 4.0.3 version.

## 3. Result

### 3.1. Transcriptomic Alternations and Functional Enrichment in COVID-19 Patients

**Figure 2:**
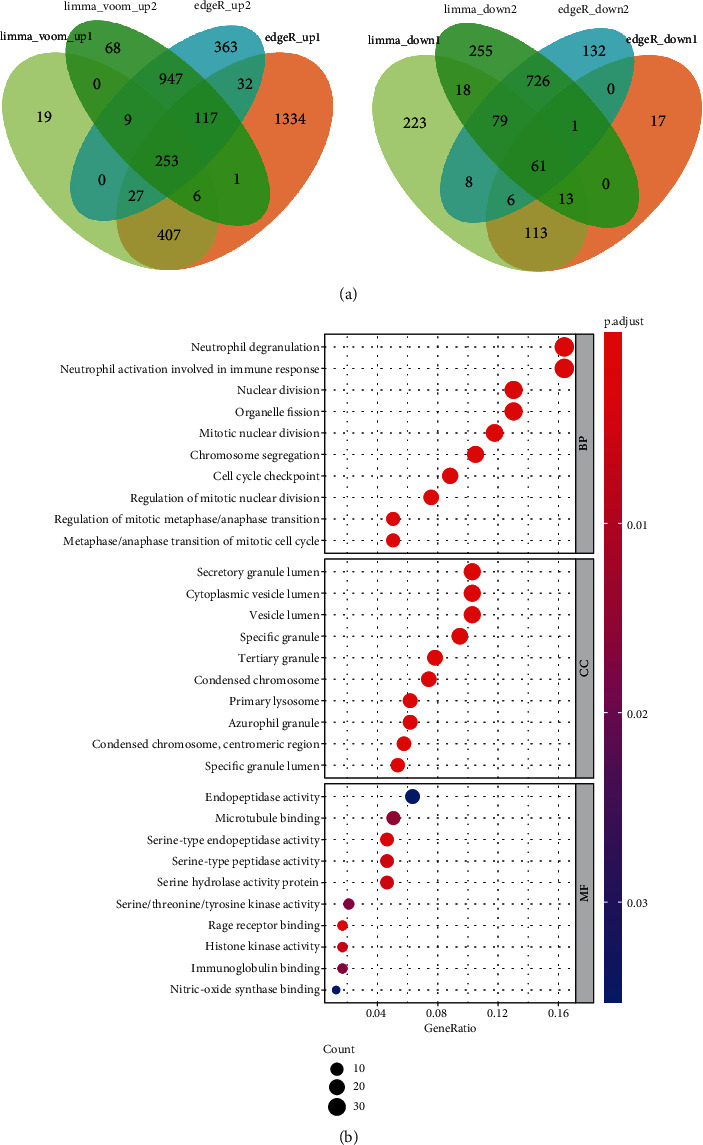
Transcriptomic alternations and functional enrichment in COVID-19 patients. (a) Differentially expressed genes (DEGs) upregulated (*n* = 278) and downregulated (*n* = 59) in COVID-19 patients compared with healthy cohort. (b) Gene Ontology analysis for the 278 upregulated genes.

Firstly, GSE152641, containing blood samples from 62 COVID-19 patients and 24 healthy controls, were obtained from the GEO database in the form of count. Then, the result of featureCounts of GSE161777 provided by the authors was merged, in which blood samples of 14 healthy people and 13 patients (first blood collection in the trial) were selected for subsequent analysis. We applied limma_voom and edgeR for each of the two datasets to increase the reliability of differentially expressed analysis. Totally, 253 genes were found to be upregulated after intersection of 4 DEG results, and 61 genes were downregulated (Figure 2(a)). GO analysis revealed that the 253 upregulated genes were enriched in the different process associated with neutrophil activation and miosis in the BP module, which was validated in the CC and MF module (Figure 2(b)). Similar results were gained in KEGG enrichment (Figure S1a). However, not significant pathways were enriched in GO and KEGG for the 61 downregulated genes (Figure S1b-c). In all, peripheral blood of COVID-19 patients might be characterized by neutrophil activation and cells were in a state of hyperproliferation.

### 3.2. Protein-Protein Interaction (PPI) Network for the DEGs

**Figure 3:**
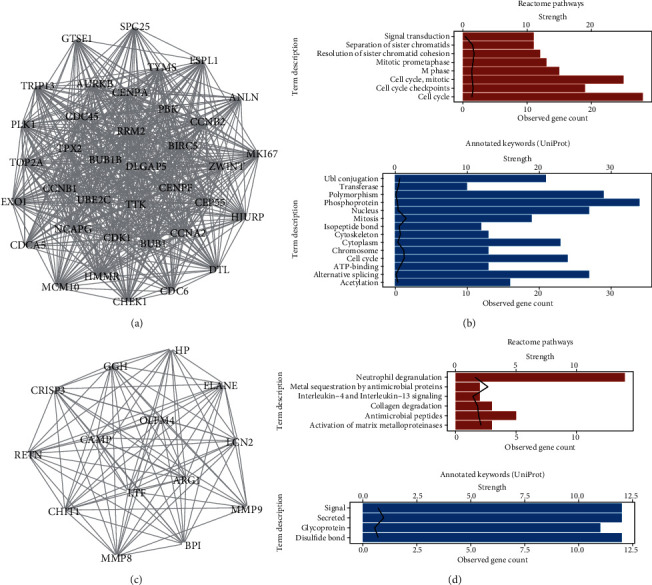
Protein-protein interaction (PPI) network for the DEGs. (a) Cluster 1 (score = 34.3, node = 37, and edge = 618) detected by molecular complex detection (MCODE) of Cytoscape. (b) Reactome pathway enrichment and UniProt database annotation for the genes in cluster1; line represented strength value. (c) Cluster2 (score = 13.5, node = 14, edge = 88) identified by MCODE. (c) Reactome pathway enrichment and UniProt database annotation for the genes in cluster2.

To explore the important genetic interaction of the occurrence of COVID-19, we utilized the STRING database to construct the PPI network of the 314 DEGs and only the genes with interaction scores larger than 0.7 were extracted, which was then put into Cytoscape. The PPI network was visualized containing 153 nodes and 1253 edges (Figure S2). The size and color of the nodes as well as edges had been mapped according to the statistic results by NetworkAnalyzer. Furthermore, MCODE was used to identify the key subnets, with all the parameters set to default. We then presented the first two significant clusters, which were again put into STRING for the functional enrichment. Genes in cluster1 (score: 34.3, 37 nodes, and 618 edges) mostly involved in the cell cycle according to Reactome Pathway enrichment; protein-annotated keyword by UniProt showed the similar results (Figures 3(a)–3(b)). Genes in cluster2 (score: 13.5, 14 nodes, and 88 edges) involved in Neutrophil degranulation, which are mostly secretory protein and signaling protein (Figures 3(c)–3(d)). Totally, there is more evidence to support that neutrophil degranulation and the strong cell proliferation status were the significant characteristics of the infection of SARS-CoV-2 (early stage).

### 3.3. Difference of Immune Cell Abundance between COVID-19 Patients and Healthy People

**Figure 4:**
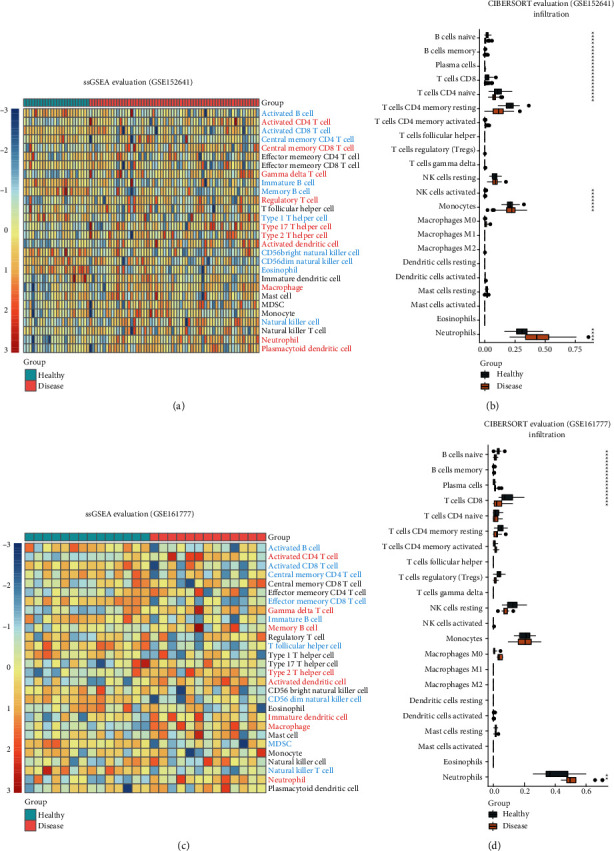
Difference of immune cell abundance between COVID-19 patients and healthy people (a) ssGSEA for evaluation of 28 immune cell infiltration in GSE152641. (b) CIBERSORT for evaluation of 22 immune cell infiltration in GSE152641. (c) SsGSEA for evaluation of 28 immune cell infiltration in GSE161777. (d) CIBERSORT for evaluation of 22 immune cell infiltration in GSE161777.

In order to clarify the alteration of infiltration of different types of immune cells in the peripheral blood, we applied ssGSEA and CIBERSORT to evaluate the immune cell abundance. For both of the two datasets, ssGSEA identified the increase of activated CD4 T cell, gamma delta T cell, type 2 T helper cell, activated dendritic cell, macrophage, and neutrophil (Wilcox test, *p* value < 0.05) in COVID-19 patients compared to healthy people. ssGSEA also identified the decrease of activated B cell, activated CD8 T cell, immature B cell, and natural killer cell (Figures 4(a) and 4(c)). As for CIBERSORT, for both of the two datasets, plasma cells, macrophages M0, and neutrophils were identified to be upregulated in COVID-19 patients significantly (Wilcox test, *p* value < 0.05) but naïve B cells; T cells CD8 were detected to be downregulated (Figures 4(b) and 4(d)). Overall, we supposed that the occurrence of COVID-19 was accompanied by activation of neutrophil and macrophage, especially neutrophil. However, there was a dramatic alteration of lymphocytes, CD 8 cells, and naïve B cells that were considered to be downregulated in the COVID-19 patients.

### 3.4. Relationship between Transcriptomic Alternations and Severity of Patients

**Figure 5:**
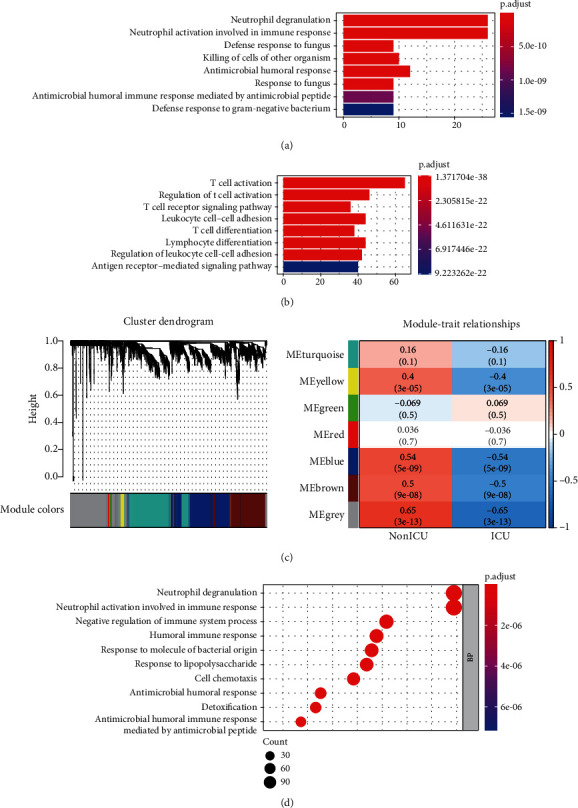
Relationship between transcriptomic alternations with severity of patients. (a) Biological process (BP) of GO analysis for genes upregulated (*n* = 67) in ICU patients compared to non-ICU patients in GSE157103. (b) BP of GO analysis for genes downregulated (*n* = 309) in the ICU patients. (c) Modules clustering and their relationship with clinical traits in WGCNA. (d) BP of GO analyses for genes in the grey module.

The transcriptomic and immune cell infiltration alternations during the occurrence of the disease have been revealed in the above study, but we wondered the immunological factors in disease progression. GSE157103, containing RNA-Seq data of peripheral blood leukocyte samples and various clinical data from 50 ICU and 50 non-ICU COVID-19 patients, was downloaded from the GEO database in the form of TPM. Limma package for DEG analysis identified 376 DEGs, including 67 upregulated genes and 309 downregulated genes, which were enriched in neutrophil degranulation and T cell activation in BP of GO, respectively (Figures 5(a) and 5(b)). To verify the DEGs, next, WGCNA was conducted on the top 5000 genes with the max median absolute deviation. 7 modules were clustered under the power value set to 30 (Figure 5(c), 1, Figure S3a). The grey module presented the highest correlation with the clinical traits (correlation efficient = 0.65) (Figure 5(c), 2). Thus, genes in the grey module were extracted for GO and KEGG analysis, which still showed that neutrophil degranulation and neutrophil activation involved in immune response played a crucial role (Figure 5(d), Figure S3).

### 3.5. Drug-Gene Interaction Analysis for Genes Related with Hospital-Free Days

**Figure 6:**
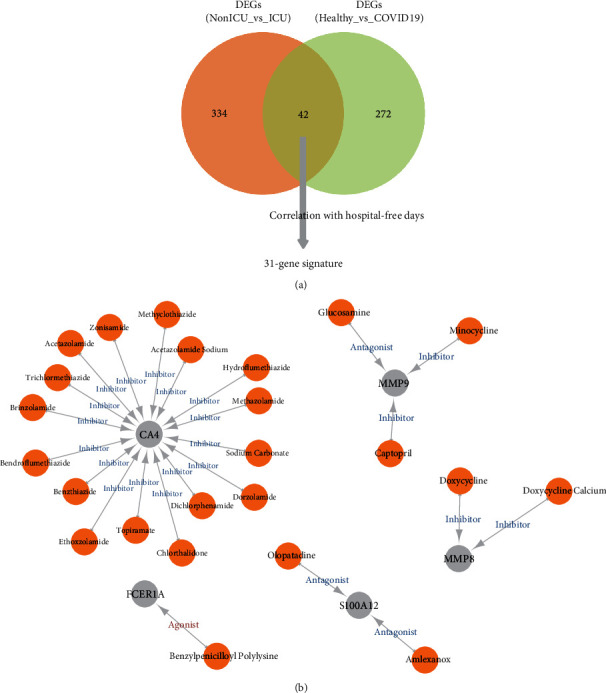
Drug-gene interaction analysis for genes related with hospital-free days. (a) Pipeline for the selection of the gene signature. (b) Drug-gene interaction predicted by DGIdb.

In order to establish a gene signature representing the occurrence and development of COVID-19, we designed a pipeline for constructing the signature (Figure 6(a)). Firstly, we intersected the 314 and 376 DEGs gained from the above analysis. The 42 genes represented the molecule made sense both in the occurrence and progress of the disease. Then, the correlation coefficients between the 42 genes and the hospital-free days during 45 days of follow-up were calculated. And 31 genes with the coefficient larger than 0.4 or smaller than −0.4 were selected, which were considered as the factors that had influence on the clinical outcome. We regarded the 31 genes as a “signature” always active in COVID-19 (Table 1).

Next, the DGIdb database was used to predict the drug-gene interaction. All of the 31 genes were input and only drugs that had been approved and had a clear pharmacological effect (inhibitor or activator) were included. Drugs targeting 5 of the 31 genes, including CA4, S100A12, MMP8, MMP9, and FCER1A were identified (Figure 6(b)). We had gotten that CA4, S100A12, MMP8, and MMP9 were related to a longer hospital day (inhibitor needed) while FCER1A was related to a longer hospital-free days (activator needed). For CA4, 16 kinds of inhibitors were found. Trichlormethiazide and bendroflumethiazide were the top two with the highest query score and interaction score. For S100A12, olopatadine and amlexanox tended to be the inhibitors. Doxycycline and doxycycline calcium were found to target MMP8, while glucosamine, minocycline, and captopril targeted MMP9, and benzylpenicilloyl polylysine can act as an agonist for FCER1A.

### 3.6. Difference of Immune Cell Abundance between ICU Patients and Non-ICU Patients and Immune Subtypes

**Figure 7:**
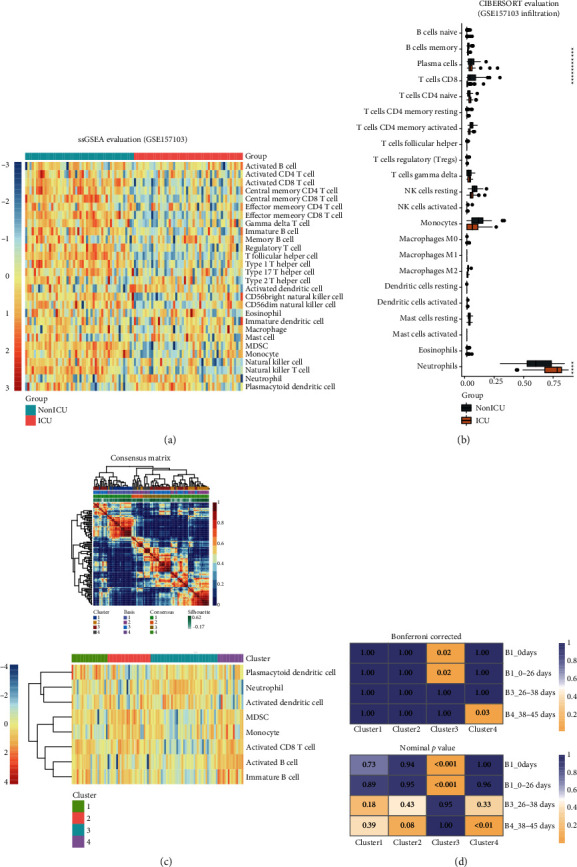
Difference of immune cell abundance between ICU patients and non-ICU patients and immune subtypes. (a) ssGSEA for evaluation of 28 immune cell infiltration in GSE157103. (b) CIBERSORT for evaluation of 22 immune cell infiltration in GSE157103. (c) 4 clusters were identified in the NMF clustering using the ssGSEA result. (d) Bonferroni-corrected and nominal *p* value of the submap result.

To explore the immunological changes during the progress of the disease, we again utilized the ssGSEA and CIBERSORT for the evaluation of immune cell infiltration on GSE157103. Interestingly, we observed a significant decline of different types of immune cells in the ICU patients based on ssGSEA. CIBERSORT also implicated the decrease of different types of immune cells, including T cell CD8 and T cell CD4 memory-activated and NK cells resting, but showed an increase of neutrophils (Wilcox test, *p* value < 0.05). Therefore, the COVID-19 patients in the ICU might show less activation of the immune system (Figures 7(a)–7(b)).

Next, to preliminarily demonstrate the impact of immune cells on clinical prognosis, we utilized the ssGSEA result to conduct the NMF clustering. 4 clusters were identified as shown in the heat map (Figure 7(c), 1). We noticed that only plasmacytoid dendritic cell, neutrophil, activated dendritic cell, MDSC, monocyte, activated CD8 T cell, activated B cell, and immature B cell were included in the clustering. Cluster3 was the subtype abundant of the first 3 cells and poor of the latter 5 cells, and cluster4 was opposite (Figure 7(c), 2). Then, patients were divided into 4 groups according to the hospital-free days during the 45 days of follow-up: 0 days (always in hospital), 0–26 days, 26–38 days, and 38–45days. Submap was applied to evaluate the similarity of gene expression characteristics between cluster1–4 and B1–4. Interestingly, the Bonferroni-corrected *p* value hinted that cluster3 (subtype of abundant plasmacytoid dendritic cell, neutrophil, and activated dendritic cell but poor of others) could be mapped to the patients with short hospital-free days (Bonferroni corrected *p* = 0.02), while cluster4 could be mapped to the patients with a relatively good prognosis (Figure 7(d)).

### 3.7. Immune Cell Abundance Was Closely Related with Hospital-Free Days and Gene Signature

**Figure 8:**
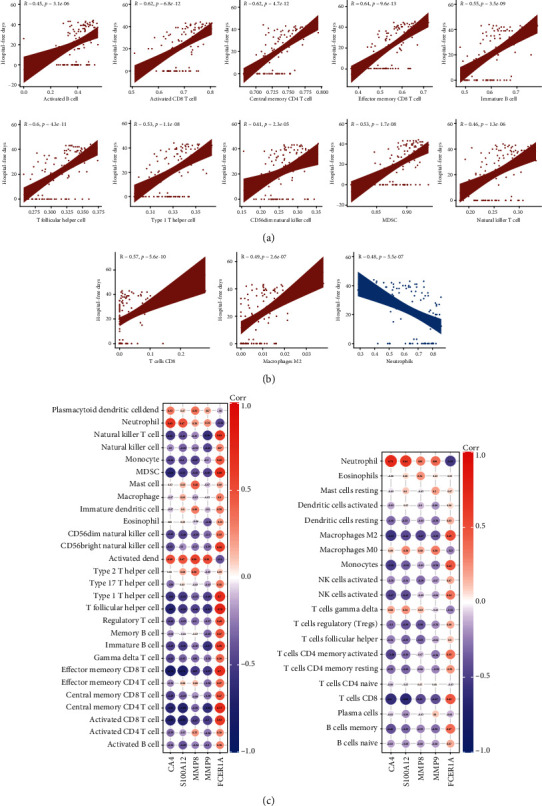
Immune cell abundance was closely related with hospital-free days and gene signature. (a) Immune cells were significantly related with hospital-free days (∣Spearman correlation coefficient∣>0.4) in ssGSEA. (b) Immune cells were significantly related with hospital-free days (∣correlation coefficient∣>0.4) in CIBERSORT. (c) Correlation between the expression level of CA4, S100A12, MMP8, MMP9, and FCER1A and immune cell infiltration evaluated by ssGSEA. (d) Correlation between the expression levels of CA4, S100A12, MMP8, MMP9, and FCER1A and immune cell infiltration evaluated by CIBERSORT.

The correlation coefficient between different types of immune cell infiltration and the hospital-free days during 45-day follow-up was calculated; ssGSEA identified 10 types of immune cells which could ameliorate the patient's hospitalization (Figure 8(a)). Unfortunately, most of them degraded in the ICU patients compared to non-ICU patients. Furthermore, CIBERSORT identified a negative impact of neutrophils on the hospital days (Figure 8(b)). Integrated with the previous analysis, it was credible that lymphocytes, especially CD8 T cells, were a protective factor of COVID-19 and the neutrophil could be a risk factor. Additionally, to understand the mechanism of the effect of the immune cells, we listed the correlation between the immune cells and the 31-gene signature as well as the correlation between the cells and the 5 genes with targeted drugs (Figure S4a-b). Whether in ssGSEA or CIBERSORT, it was implicated that CA4, S100A12, MMP8, and MMP9 were related with the regression of lymphocytes, especially CD8 T cells, while related with the activation of neutrophil. Conversely, FCER1A was related to the activation of various kinds of immune cells but was negatively correlated with neutrophil infiltration (Figure 8(c)). In short, studies at the level of transcriptome and immune cells can be integrated, which also provided an explanation for the predicted drugs.

## 4. Discussion

Although the pandemic of COVID-19 has threatened the health of the world, the host immune response to SARS-CoV-2 infection still lacks full demonstration. Up to now, evidence showed that an imbalanced immune response to inflammation is a major trigger of COVID-19 and the dysfunction of local and systemic immune responses had been implicated in the disease outcome and prognosis. Thus, identifying transcriptomic and immunological alternations may not only be significant for a better comprehensive understanding of the mechanisms of the disease but also help to effective therapy excavation and individualized management.

In the present study, we paid close attention to both the occurrence (comparison1: healthy vs. patients) and the progress (comparison2: non-ICUers vs. ICUers) of COVID-19. 253 upregulated genes and 61 downregulated genes were identified to be differentially expressed during the occurrence of the disease. GO, KEGG, Reactome, and UniProt were used to annotate the function of DEGs, and the PPI network was constructed, with 2 crucial subnets identified. WGCNA was used to find the significant gene modules. ssGSEA and CIBERSORT revealed that neutrophil activation and CD8+ T cell downregulations were two reliable changes in both of the comparisons. Novelly, several drugs were predicted and the pharmacological effects were understood.

Combined with the enrichment result and the evaluation of immune cells, it was clear that peripheral blood of the COVID-19 patients was in a state of hyperproliferation and immune cells show overall activation. Such changes had been considered to be strongly related to oxidative stress, which strengthened the immune system but could also cause excessive inflammatory and respiratory failure [49, 50]. Inferred from two comparisons, the beneficial immune defense gradually transformed into an excessive inflammatory response, while the immune system would be in a state of exhaustion. On the other hand, neutrophils, which was believed to play a key role during the disease course in the present study, could also lead to damage through oxygen species (ROS) [51].

Here, we will review the genes that had potential drugs, which owned a close relationship with oxidative stress and inflammation. CAs, which catalyze the interconversion of water and carbon dioxide into dissociated ions of carbonic acid, are a kind of zinc metalloenzymes broadly engaged in various biological processes [52–54]. There are 14 isozymes of CAs altered genetically in the pathological status in human [55], and CA4 is the most widely distributed one [56]. CA4 plays an important role in the bicarbonate reabsorption of the kidney [57]. During acidosis, its competence is enhanced to generate more H+ to relay the acidosis [58]. We assume that CA4 on blood cells can act likely in the acidotic status resulting from hypoxia created by COVID-19. And CA4 may affect the function of neutrophils by modulating altering pH [59].

Next, MMPs are an enzyme family majorly correlated with the remodeling of extracellular matrix (ECM) components [60]. MMP9 (or gelatinase B) is one of the main types of MMPs and can be found in diverse cells like monocytes, macrophages, and neutrophils. MMP9 are highly expressed in pathological processes including inflammation [61], as is also discovered in this study. MMP9 is an inflammatory cytokine, acting as a regulator to promote the secretion of other cytokines by leukocytes. Besides, MMP9 itself is also regulated by the degranulation from neutrophils, which is induced by other various types of chemotactic factors. Moreover, MMP9 can truncate IL-8, the major human neutrophil chemoattractant, into a tenfold more potent form, creating a positive feedback loop for neutrophil activation and chemotaxis [62]. MMP8 is majorly synthesized and archived in neutrophils [63]. Circulating MMP8 has been found to closely related with lung fibrosis in COVID-19 patients [64] and serves as member of a 5-protein classifier to predict the prognosis of idiopathic pulmonary fibrosis (IPF) [65].

Besides, S100A12 is a member of the S100 protein family of calcium-binding ability and is predominantly secreted by neutrophils [66, 67]. As an emerging biomarker for inflammatory diseases, the level of S100A12 in serum can reflect the systemic inflammatory status in acute otitis media, cystic fibrosis, respiratory distress syndrome, and dermatomyositis-associated interstitial lung disease [67, 68]. Besides, S100A12 is also found to herald worse cardiac output and mortality in pulmonary hypertension [69], which is also common in COVID-19 [70]. Moreover, SA100A12, together with S100A8 and S100A9, which are also both released by neutrophils, can activate airway epithelial cells to produce MUC5AC, a major mucin protein in the respiratory tract [71], partly interoperating the excessive mucus discovered in the necropsy of COVID-19 patients [72]. And compared with S100A8 and S100A9, SA100A12 is more considered as a marker for respiratory diseases with neutrophilic inflammation [73].

Furthermore, FCER1A encodes a subunit of Fc*ε*R that can bind with IgE [74] and can be found on the surface of various kind of cells like basophils, mast cells, monocytes, dendritic cells, and neutrophils [75]. Studies looking into FCER1A in the respiratory system mainly focus on mast cell and basophils, two major cells involved in allergy [76]. But the decreased level of FCER1A in COVID-19 patients seemed to not explain the potential role of the activation of mast cell or that basophil plays in hyperinflammation with patients [77, 78], which deserves to be further studied.

With regard to the immune cell infiltration of the blood, the elevation of the abundance of neutrophils is universally observed in various studies [79]. This study also suggested the important role of neutrophils in the pathological process of COVID-19. Infected lung cells are found to express neutrophil-attracting chemokines, and attracted neutrophils can attract even more neutrophils that might finally result in the excessive activation and degranulation of neutrophils, contributing to neutrophil-related lung damage [80, 81]. Several possible mechanisms concerning neutrophils are proposed. Neutrophil extracellular traps (NETs), which refer to web-like chromatin structures derived from dead neutrophils [82], might be one of the most prevalent ones [83]. MMP8, MMP9, and S100A12, three genes that we found significantly upregulated in COVID-19 patient, are also common components in NETs [84–86], which further demonstrates the vital roles of NETs in COVID-19 development.

On the other hand, the abundance of CD8+ T cells was reported to decrease in COVID-19 patients and exhibit functional exhaustion molecules, such as NKG2A, PD-1, and TIM-3 [87]. And neutrophil-to-lymphocyte can also increase as a result of systemic inflammation serving as a prognostic marker [88]. Single-cell RNA sequencing of bronchoalveolar cells depicted a more complicated landscape of CD8+ T cells in COVID-19 patients, further pointing out the heterogeneity of cell numbers and clonal expansion of different CD8+ T cell clusters [89].

Herein, we can understand the mechanisms of the drugs which were predicted in the present study based on the above discussion. Trichlormethiazide and bendroflumethiazide are both inhibitors for CAs [90, 91]. Application of CA inhibitors in COVID-19 individuals can block the discharge of H+ in the kidney and worsen the acidotic status in patients [92]. Besides, application of the CA inhibitor can rescue the decrease of IL-8, the most important chemotactic for neutrophils in hypercarbia, which might deteriorate the overactivation of neutrophils [59]. Olopatadine is an antiallergic drug antagonizing the histamine H(1) receptor [93]. Amlexanox is a small-molecule targeted therapy used to treat atopic diseases [94]. Both olopatadine and amlexanox were found to have the ability to suppress the migration of monocytes induced by proinflammatory S100A12 [95]. Doxycycline has antimicrobial effect as well as potent anti-inflammatory activity [96]. And doxycycline can downregulate MMP8 both in mRNA and protein levels [97]. Minocycline is another kind of common antibiotic used in bedside and it was found to reduce the level of MMP9 [98, 99]. Captopril is one of angiotensin-converting enzyme inhibitors (ACEIs) usually used to relieve hypertension [100] and can also downregulate the expression of MMP9 and reactive oxygen species (ROS) [101].

## 5. Conclusion

Based on 3 dependent RNA-seq of COVID-19 patients, we learned that the neutrophil degranulation was significant in the occurrence of the disease, during which the peripheral blood was in a hyperproliferative state. Neutrophil activation and the inactivation of CD8+ T cells played a key role during the progress of the disease and 4 immune subtypes were identified. A 31-gene composed signature was established which was crucial during the course of the disease. Several drugs were predicted for the therapies of COVID-19 based on the prognostic value of the genes in the signature. In short, we believe that our study shed light on the understanding and treatment of COVID-19.

## Figures and Tables

**Table 1:** 31 Genes in the signature of COVID-19.

Gene	Correlation coefficient	*p* value
*PID1*	0.693520919	1.27*E*−15
*P2RY10*	0.679852476	7.38*E*−15
*CD40LG*	0.668959371	2.81*E*−14
*FCER1A*	0.654672468	1.49*E*−13
*CD5*	0.645849101	4.00*E*−13
*TCF7*	0.636922512	1.05*E*−12
*FAM102A*	0.624610777	3.80*E*−12
*TRABD2A*	0.623338096	4.32*E*−12
*NELL2*	0.618426883	7.08*E*−12
*CPA3*	0.593173164	7.88*E*−11
*TPPP3*	0.584820798	1.67*E*−10
*HDC*	0.57412751	4.24*E*−10
*MAL*	0.536815499	8.54*E*−09
*PRSS33*	0.521039587	2.73*E*−08
*ALOX15*	0.516734511	3.72*E*−08
*VSIG4*	−0.429548434	8.21*E*−06
*MMP8*	−0.472200179	7.05*E*−07
*ANXA3*	−0.517766599	3.46*E*−08
*CHIT1*	−0.529930553	1.43*E*−08
*ADAMTS2*	−0.53487937	9.89*E*−09
*PCOLCE2*	−0.544461309	4.76*E*−09
*TPST1*	−0.551635666	2.71*E*−09
*WFDC1*	−0.555936581	1.92*E*−09
*IL18R1*	−0.561055133	1.27*E*−09
*CA4*	−0.581762072	2.19*E*−10
*MMP9*	−0.583963653	1.80*E*−10
*CD177*	−0.594606481	6.91*E*−11
*ARG1*	−0.60433151	2.79*E*−11
*OLAH*	−0.619818119	6.16*E*−12
*S100A12*	−0.646161289	3.87*E*−13
*MCEMP1*	−0.683772837	4.50*E*−15

## Data Availability

Three datasets were obtained publicly from the Gene Expression Omnibus (GEO) database (https://www.ncbi.nlm.nih.gov/geo/); further inquiries for the codes or other data can be directed to the corresponding author.

## Supplementary Materials

Supplementary Materials The Supplementary Material for this article can be found online. Supplementary Figure 1: functional enrichment for the DEGs (a) KEGG analysis for the 253 up-regulated genes. (b) GO analysis for the 61 downregulated genes. (c) KEGG analysis for the 61 downregulated genes. Supplementary Figure 2: the PPI network for the total 314 genes. Supplementary Figure 3: WGCNA for the top 5000 genes with the max median absolute deviation. (a) Selection for the best power value. The *R*^2^ threshold was set to 0.8. (b) KEGG analysis for the genes in grey module. Supplementary Figure 4: correlation between genes in the signature and immune cells. (a) Correlation between the 31 genes in the signature and 28 immune cells evaluated by ssGSEA. (b) Correlation between the 31 genes in the signature and 22 immune cells evaluated by CIBERSORT.
