# Supplementary material for: Comprehensive Analysis of the Systemic Transcriptomic Alternations and Inflammatory Response during the Occurrence and Progress of COVID-19
Source: Oxid Med Cell Longev. 2021 Aug 26;2021:9998697. doi: 10.1155/2021/9998697 (PMC8397550; doi:10.1155/2021/9998697)

Supplementary materials

(Supplementary Figure 1-4)

Supplementary Figure 1 (a) KEGG analysis for the 253 up-regulated genes. (b) GO analysis for the 61 down-regulated genes. (c) KEGG analysis for the 61 down-regulated genes.

Supplementary Figure 2 PPI network for the total 314 genes

Supplementary Figure 3 (a) Selection for the best power value. R^2 threshold was set to 0.8. (b) KEGG analysis for the genes in grey module.

Supplementary Figure 4 (a) Correlation between the 31 genes in the signature and 28 immune cells evaluated by ssGSEA. (b) Correlation between the 31 genes in the signature and 22 immune cells evaluated by CIBERSORT.

Supplementary Figure 1

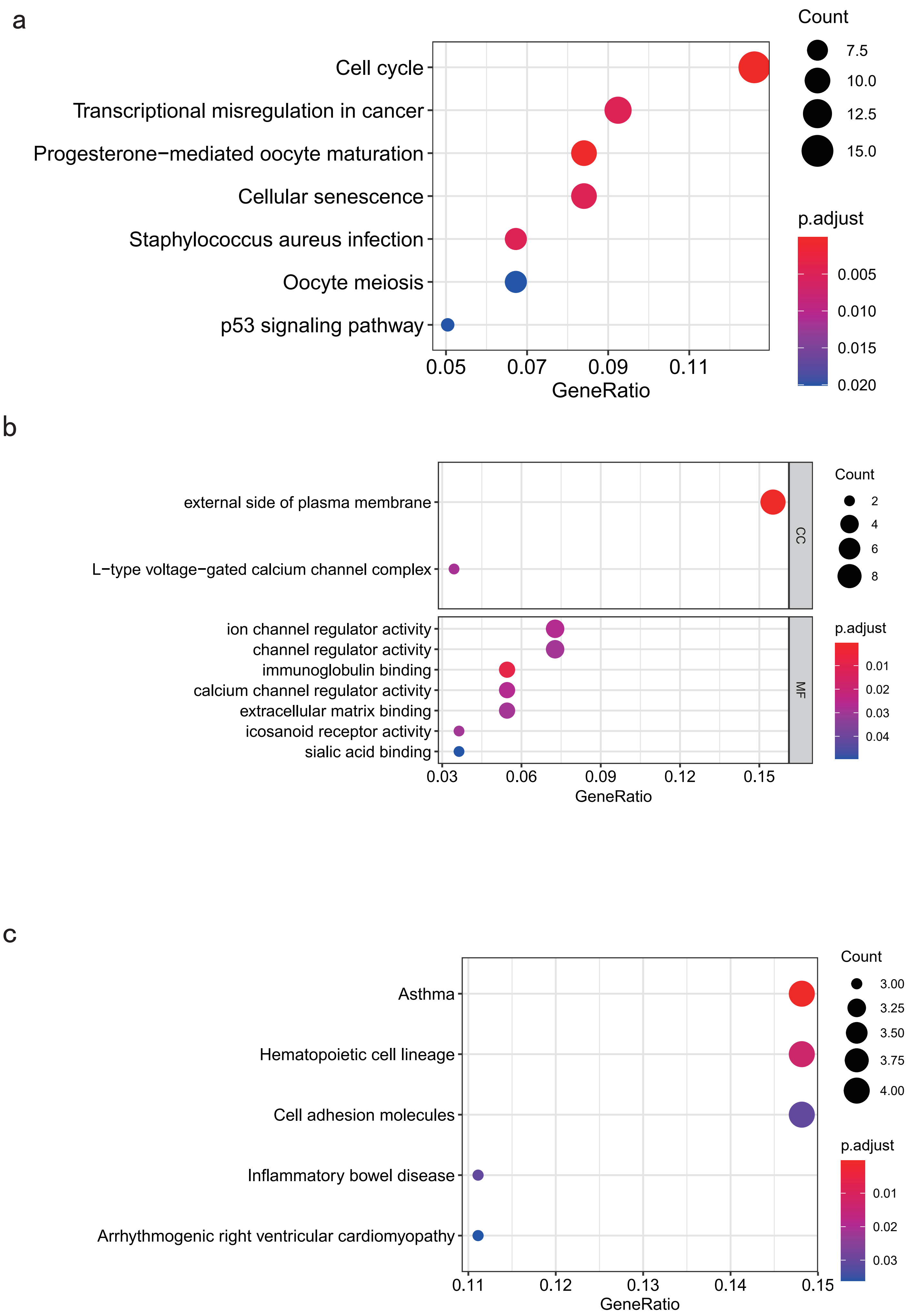

Supplementary Figure 2

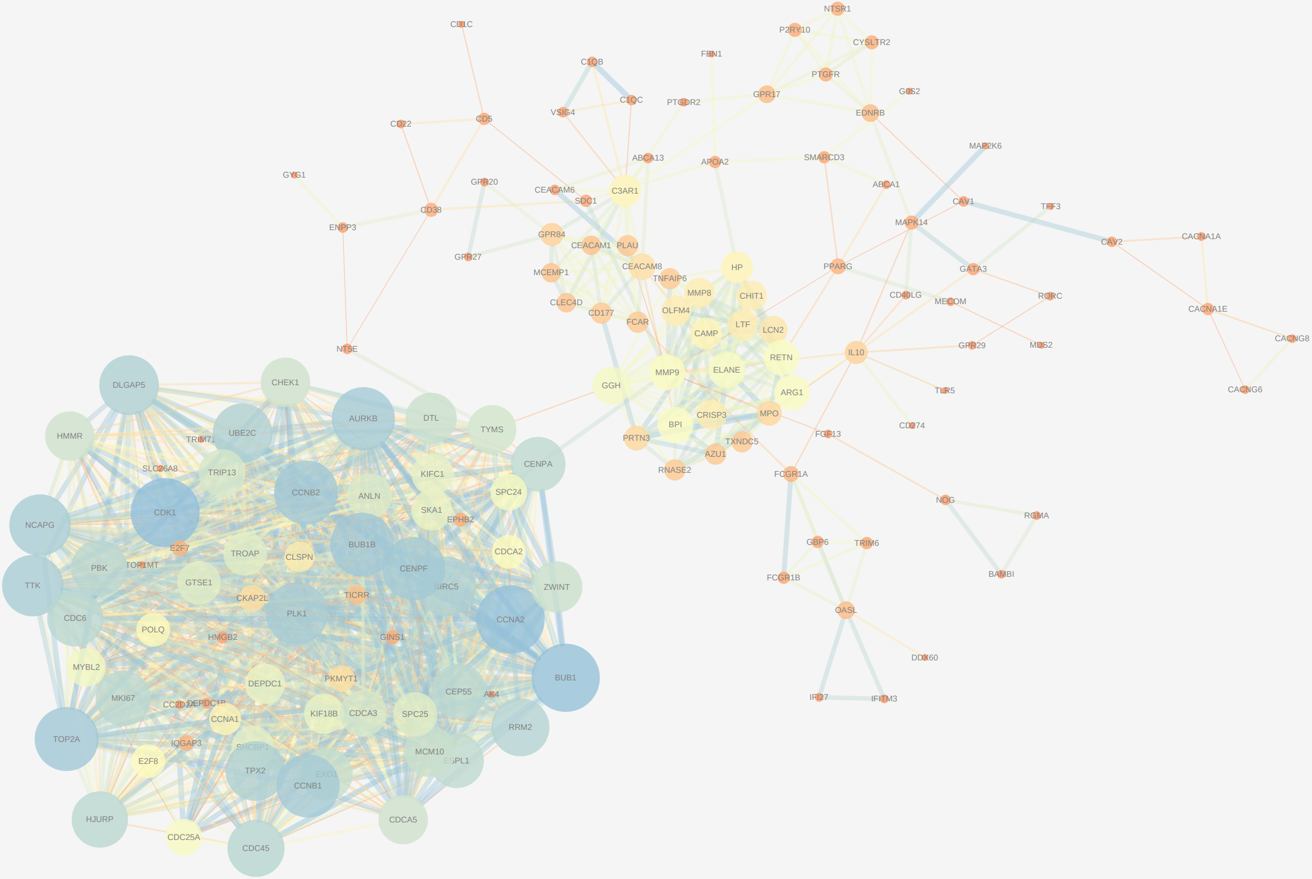

Supplementary Figure 3

a

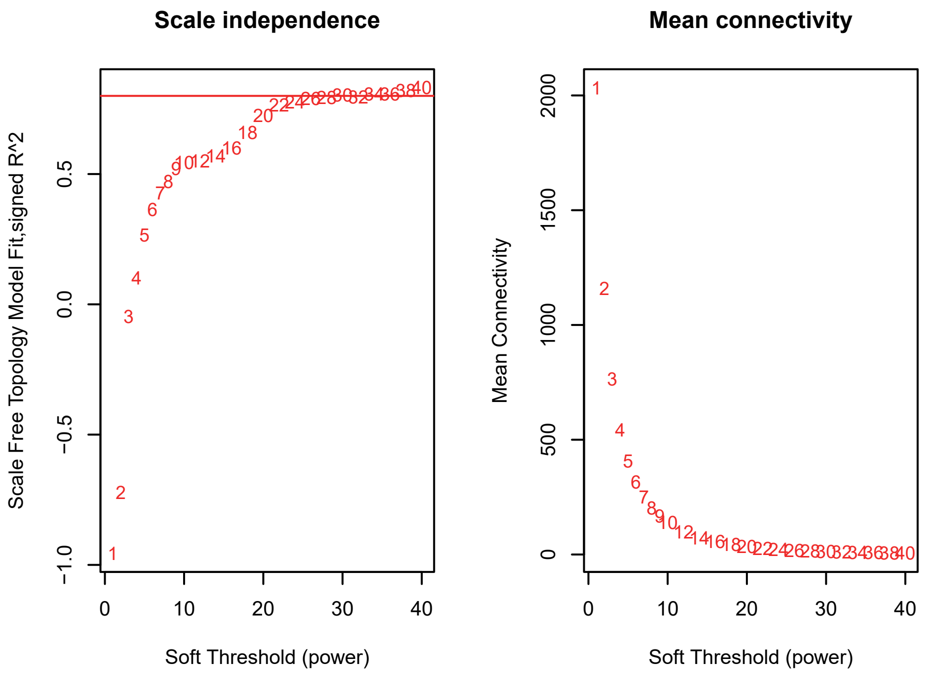

b

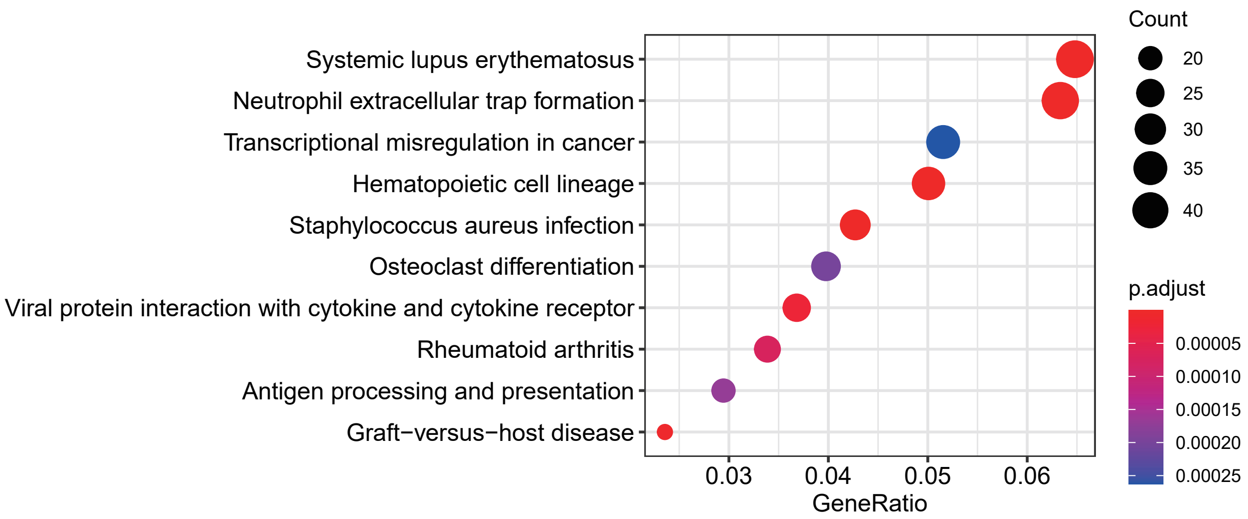

Supplementary Figure 4

a

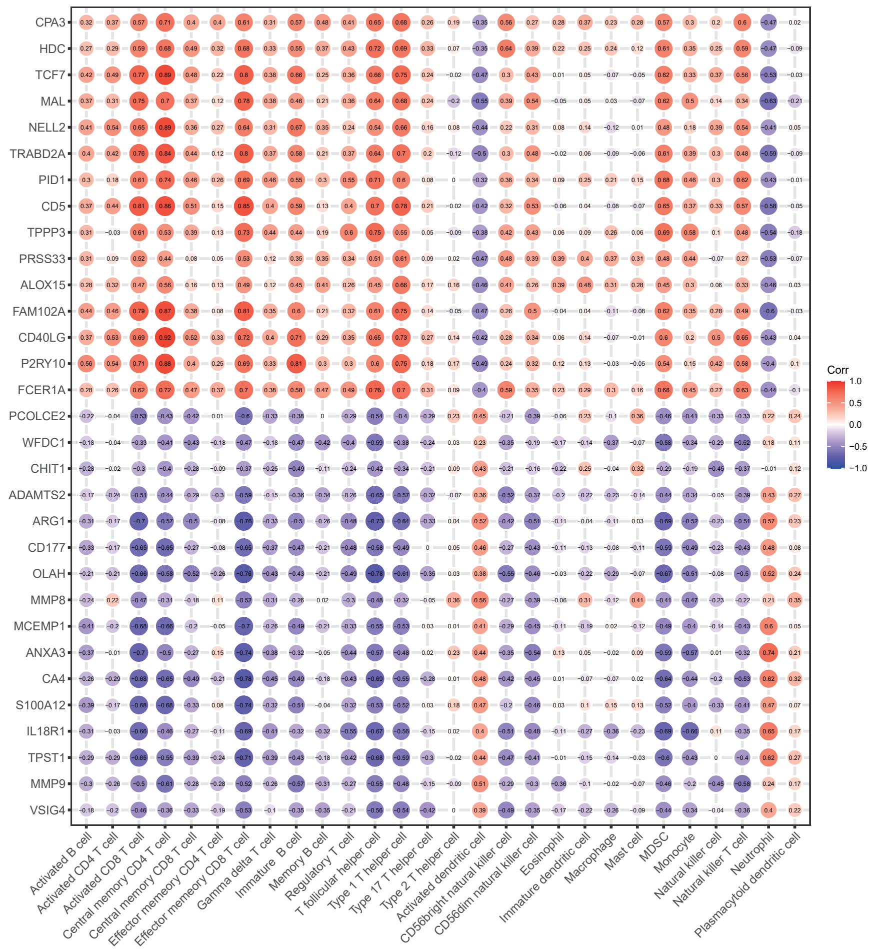

b

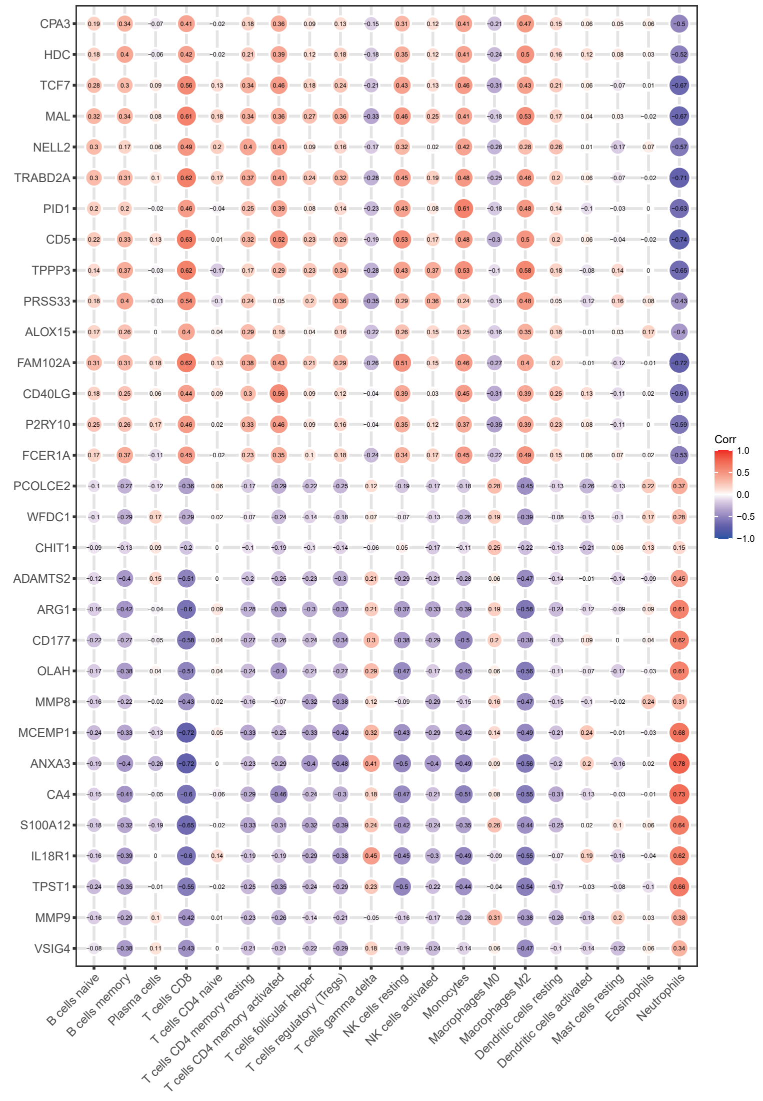

Supplement: Supplementary Materials — The Supplementary Material for this article can be found online. Supplementary Figure 1: functional enrichment for the DEGs (a) KEGG analysis for the 253 up-regulated genes. (b) GO analysis for the 61 downregulated genes. (c) KEGG analysis for the 61 downregulated genes. Supplementary Figure 2: the PPI network for the total 314 genes. Supplementary Figure 3: WGCNA for the top 5000 genes with the max median absolute deviation. (a) Selection for the best power value. The R2 threshold was set to 0.8. (b) KEGG analysis for the genes in grey module. Supplementary Figure 4: correlation between genes in the signature and immune cells. (a) Correlation between the 31 genes in the signature and 28 immune cells evaluated by ssGSEA. (b) Correlation between the 31 genes in the signature and 22 immune cells evaluated by CIBERSORT. [file 9998697.f1.pdf]
